# Dexmedetomidine as a Procedural Sedative for Percutaneous Tracheotomy: Case Report and Systematic Literature Review

**DOI:** 10.1155/2012/659415

**Published:** 2012-11-29

**Authors:** Jerrold L. Perrott, Michelle T. Co, Steven C. Reynolds, Derek J. R. Gunning

**Affiliations:** ^1^Departments of Pharmacy and Critical Care, Royal Columbian Hospital, New Westminster, BC, Canada; ^2^Faculty of Pharmaceutical Sciences, University of British Columbia, Vancouver, BC, Canada; ^3^Department of Pharmacy, Ridge Meadows Hospital, Maple Ridge, BC, Canada; ^4^Department of Critical Care, Royal Columbian Hospital, New Westminster, BC, Canada; ^5^Department of Medicine, University of British Columbia, Vancouver, BC, Canada; ^6^Departments of Cardiac Surgery and Critical Care, Royal Columbian Hospital, New Westminster, BC, Canada

## Abstract

*Purpose*. To describe the successful use of dexmedetomidine as the primary procedural sedative for a percutaneous tracheotomy procedure and to systematically present the supporting literature. *Materials and Methods*. A Case report of our experience and systematic literature search. PubMed, Embase, and Google Scholar were searched without restriction using the key words *dexmedetomidine*, *percutaneous tracheotomy*, and *tracheotomy procedure*. All relevant published references were retrieved irrespective of their methodological quality. *Results*. In total, only 3 relevant references were found. These include one small placebo controlled randomized trial and 2 case reports. The randomized, placebo controlled trial enrolled patients already sedated on midazolam and included 64 total patients. The 2 other case reports both described the use of dexmedetomidine as the primary procedural sedative. All of the cases reported the successful completion of the percutaneous tracheotomy without any major complication, but none reported the subjective patient experience. *Conclusion*. Based on the available published literature and our experience, we suggest that dexmedetomidine be considered for use as the primary procedural sedative for percutaneous tracheotomy procedure. Dexmedetomidine's ability to provide adequate sedation and amnesia, without blunting the respiratory drive and protective reflexes of the patient, may make it an optimal agent in specific cases.

## 1. Introduction

Dexmedetomidine is a centrally acting alpha-2 agonist with licensed indications for an intensive care unit sedation and a conscious sedation for Monitored Anaesthesia Care and Awake Fiberoptic Intubation. Its pharmacology is such that it provides sedative, analgesic, and antishivering effects without attenuating respiratory drive and provides a state of “cooperative sedation” whereby the patient is sedated, but still able to interact with the healthcare team [1]. The primary adverse effects observed with the procedural use are bradycardia and hypotension [1]. Other agents for procedural sedation, such as benzodiazepines and propofol, can induce significant respiratory depression and loss of protective airway reflexes, making them suboptimal in certain clinical scenarios.

The properties of dexmedetomidine make it a viable option for procedural sedation when it is vital to maintain the respiratory drive of the patient. We present a case of a percutaneous tracheotomy performed under sedation with dexmedetomidine and fentanyl with lidocaine for local anaesthesia and a systematic literature review of the evidence supporting this indication.

## 2. Case Report

An 83-year-old male, who weighed 67 kg and was 170 cm tall, presented to our hospital in the spring of 2012 with a non-ST elevation myocardial infarction and congestive heart failure. He was referred for coronary artery bypass grafting (CABG) by the interventional cardiology after an angiogram demonstrated coronary disease not amenable to percutaneous intervention. Specifically, he had a significant triple-vessel disease which had progressed in the context of two previous angiograms with the placement of coronary stents in 1997 and 2000. 

His past medical history included hypertension, dyslipidemia, a remote history of smoking, and of note, an episode of aspiration (of a carrot) at age 2 years that required a tracheostomy. Subsequent to his tracheostomy, multiple airway surgeries were completed over the following 8 years that resulted in significant supra and subglottic airway stenosis, as demonstrated on bronchoscopy approximately 10–15 years ago. As this stenosis did not impair his exercise tolerance, he elected to not pursue any corrective measures at that time.

Due to his airway concerns, anaesthesiology took special precautions in attempting to intubate the patient for his coronary artery bypass grafting. Despite multiple complex manoeuvres and the assistance of multiple airway experts, oral endotracheal intubation was unsuccessful and the process was abandoned. He was admitted to our intensive care unit awake and alert for observation on oxygen via nasal prongs at 3 L/min, with saturations of 99%. 

After discussions between the cardiac surgery, interventional cardiology, anaesthesiology, and critical care teams and with consent from the patient, it was decided that an awake percutaneous tracheotomy would the best option to facilitate progression to CABG. 

Owing to the complex airway issues, this was deemed a high risk procedure leading to the careful selection of the procedural sedation. Typical agents such as benzodiazepines and neuromuscular blocking agents were deemed unsuitable as they would impair the patient's respiratory drive and protective reflexes, especially in the event of an unsuccessful procedure. The pharmacological characteristics of dexmedetomidine were felt to provide the optimal effects to safely perform the percutaneous tracheotomy with the least risk. Specific aspects of dexmedetomidine that made it an excellent fit for this procedure were the sedative, amnestic, and opiate sparing effects, with the maintenance of respiratory drive and protective airway reflexes.

After confirming consent, the patient was prepared for tracheotomy. Preoxygenation was undertaken by administering 100% oxygen via face mask. Viscous lidocaine was applied topically to patient's tongue to reduce discomfort and gagging and upper airway topical anesthesia was obtained with atomized 2% lidocaine. A loading dose of dexmedetomidine 1 mcg/kg was infused intravenously (IV) over 20 minutes, followed by a maintenance infusion at a rate of 0.5 mcg/kg/hour. The prolonged loading time was used to reduce the likelihood of adverse cardiac effects developing. A bolus of 25 mcg of fentanyl IV was also administered. At this point, the patient was well sedated, but rousable to voice or physical stimulation, and was maintaining his airway and breathing spontaneously.

Upon the completion of the loading dose, the patient was put in the supine position with neck extension supported by a bolster under the upper torso. The larynx and previous tracheostomy sites were identified. A video bronchoscope was inserted into the airway and positioned immediately below the larynx. At this point, the patient was noted to be uncomfortable and coughing, so the maintenance infusion of dexmedetomidine was increased to 0.8 mcg/kg/hr, and an additional 25 mcg of IV fentanyl was administered. 

The position of the bronchoscope was confirmed with the transillumination of the airway and with the manipulation of the trachea. The anterior neck was prepared with chlorhexidine/alcohol solution. A site was chosen in the midline in the previous tracheostomy scar well above the manubrium and 1-2 cm below the cricoid cartilage (approximating the 3rd tracheal ring which was not clearly identifiable). The Ciaglia Blue Rhino percutaneous tracheostomy set was used. This entailed airway access with a 16-gauge needle, introduction of a J tipped guide wire via the needle, serial dilation of the tract, and insertion of a no. 6 Shiley cuffed tracheostomy tube loaded on the tracheostomy loading catheter. Bronchoscopic guidance was maintained throughout each step. The bronchoscope was then inserted into the airway via the tracheostomy tube confirming its position. Following the insertion of the tracheostomy tube, the cuff was inflated, the inner cannula was inserted, and ventilation was initiated. Adequate oxygenation and carbon dioxide clearance were confirmed. The tube was secured in place with two 0 silk sutures and tracheostomy ties. Finally, the patient was allowed to emerge from sedation.

The patient's hemodynamic measurements were monitored continuously throughout the procedure and are presented in Table 1. As expected a small degree of hypotension and bradycardia evolved and were easily managed with an IV infusion of norepinephrine. There were no episodes of oxygen desaturation.

A few hours after the procedure, once he was fully recovered from the sedation, the patient reported that he did not recall the procedure, nor recall or feel any discomfort. He was very pleased with the whole procedure and was looking forward to having his coronary artery bypass grafting performed. When approached four days after his tracheotomy, he did not recall having had a conversation with the authors on the day of his procedure.

Two weeks following the insertion of the tracheostomy, when operating room time was next available, the patient underwent coronary artery bypass via lower hemisternotomy. This access was chosen to maintain the separation of the tracheostomy and sternotomy incisions. The perioperative course was uncomplicated. The patient was discharged home with plans for urgent otolaryngology followup for the management of his supraglottic stenosis.

## 3. Materials and Methods

The Fraser Health Authority Research and Ethics Board considers a case report to be a medical/educational activity and therefore, Fraser Health researchers are not required to obtain ethics approval for such publications as long as a written, informed consent is obtained from the patient, as was done in this case.

For the literature search, PubMed, Embase, and Google Scholar were systematically searched up to May 1st, 2012 without restrictions using the terms *dexmedetomidine, percutaneous tracheotomy, *and* tracheotomy procedure*. Titles and abstracts were screened by 1 author (J. L. Perrott) and relevant full text articles were obtained. Only articles describing the use of dexmedetomidine as sedation for tracheotomy procedures, but not other airway surgical interventions or manoeuvres, were included.

## 4. Results

Three articles [2–4] were found to evaluate or describe the use of dexmedetomidine for a procedural sedation for a percutaneous tracheostomy. 

Alhashemi et al. [2] reported in abstract only the results of a small randomized placebo controlled trial. They enrolled 64 intensive care unit patients already on the intermittent midazolam sedation to either a dexmedetomidine 1 mcg/kg or saline-placebo IV bolus over 10 minutes in addition to fentanyl 1 mcg/kg IV bolus just prior to skin incision. Their primary outcome was the amount of rescue propofol bolus needed during the procedure. They demonstrated no significant differences between the dexmedetomidine and placebo groups (median 40 mg versus 45 mg, *P* = 0.7). Their only positive secondary outcome was the median time to complete the tracheotomy procedure (dexmedetomidine 7 minutes versus placebo 10 minutes, *P* = 0.04). No details of the patients' experiences of the procedures were published.

David and de Marchi [3] reported a successful awake surgical tracheostomy procedure done in a 44-year-old woman with significant neck, upper airway, and facial swelling due to local after resection and chemoradiation therapy for a metastatic tonsillar squamous cell carcinoma. She received a loading dose of dexmedetomidine of 0.5 mcg/kg IV over 10 minutes followed by an IV infusion which was titrated from 0.2 mcg/kg/hour to 0.7 mcg/kg/hour for the procedure. They also administered a local anaesthetic with lidocaine 1% and a supplemental propofol 20 mg IV bolus for sedation during the initial airway instrumentation. Their patient had one episode of transient desaturation (down to an S_a_O_2_ of 86%), but no other complications were noted. They did not report any details of the patient's subjective experience.

Finally, Kunisawa et al. [4] reported a successful percutaneous tracheotomy procedure completed in a ninety-year-old 44 kg male who was one week after the evacuation of an intracranial hematoma. Clinically he had a persistently decreased level of consciousness and left hemiplegia. He was administered a dexmedetomidine 1 mcg/kg IV loading dose over 10 minutes, followed by an infusion of 0.7 mcg/kg/hour. Local anaesthesia with 1% lidocaine was also administered. They reported no complications during their procedure and did not report details of the patient's subjective experience.

## 5. Discussion

We present a case where dexmedetomidine was used successfully for a percutaneous tracheotomy in an 83-year-old male with a history of sub- and supraglottic airway stenosis. Adequate procedural sedation was maintained for completion of the procedure and for patient comfort and amnesia. The protracted period of amnesia after the full recovery of the level of consciousness was unexpected and should be examined in future studies to better characterize its total duration.

The literature reported on this topic is sparse and of overall low methodological quality [5]. The only randomized controlled trial was completed in patients already sedated with midazolam, whereas the case reports are more similar to the patient we present. Ideally, a controlled trial would be conducted to better define the place of dexmedetomidine for use as the primary procedural sedative for percutaneous tracheotomy procedures; however, this is unlikely to be completed. Future publication of further observational cases and data would, at the least, serve to better support this use for dexmedetomidine.

## 6. Conclusions

Dexmedetomidine in conjunction with small bolus dose narcotic provides adequate procedural sedation to complete percutaneous tracheotomy procedures without inhibiting the patient's protective airway reflexes or respiratory drive. The hemodynamic effects that can occur with dexmedetomidine can be easily managed with the judicious administration of vasopressors. Dexmedetomidine may induce an amnestic response, both during and for a number of hours after the infusion.

Based on our experience and the published literature of other similar cases, we recommend considering dexmedetomidine as the primary sedative for use during percutaneous tracheotomy procedures in patients for whom other agents are inappropriate or of a higher risk, though future research on this topic is necessary to fully characterize the efficacy and safety of dexmedetomidine for this use.

We suggest that dexmedetomidine be dosed following the manufacturers recommended administration for “Awake Fiberoptic Intubation” [6] with a loading dose of 0.5–1 mcg/kg IV over 10 minutes (with the lower dose used for elderly patients) followed by an infusion of 0.2–1 mcg/kg/hour IV, starting at 0.6 mcg/kg/hour. Careful monitoring of the vital signs and level of consciousness should be undertaken throughout the induction, maintenance, and recovery phase of the procedure.

## Figures and Tables

**Table 1:** Vital signs and infusions during tracheotomy procedure.

Time	HR	SBP	DBP	MAP	Dex. dose	NE dose	Comments
10:40	55	155	50	85	3	0	Dexmedetomidine load started
10:45	50	145	50	81	3	0	
10:50	55	115	45	68	3	2	
10:55	50	115	45	68	3	2	Dexmedetomidine load completed
11:00	50	135	50	78	0.5	2	Bronchoscope inserted
11:05	55	115	45	68	0.8	2	
11:10	50	130	55	80	0.8	0	
11:12	70	155	70	98	0	0	Procedure completed

DBP: diastolic blood pressure (mmHg); Dex. dose: dexmedetomidine infusion rate (mcg/kg/hr); HR: heart rate (beats per minute); MAP: mean arterial pressure (mmHg); NE dose: norepinephrine infusion rate (mcg/min); SBP: systolic blood pressure (mmHg).
